# Learning unfamiliar pitch intervals: A novel paradigm for demonstrating the learning of statistical associations between musical pitches

**DOI:** 10.1371/journal.pone.0203026

**Published:** 2018-08-30

**Authors:** Yvonne Leung, Roger Thornton Dean

**Affiliations:** MARCS Institute for Brain, Behaviour and Development, Western Sydney University, Sydney, New South Wales, Australia; University of Zurich, SWITZERLAND

## Abstract

While mastering a musical instrument takes years, becoming familiar with a new music system requires less time and skills. In this study, we examine whether musically untrained Western listeners can incidentally learn an unfamiliar, microtonal musical scale from simply engaging in a timbre discrimination task. The experiment is comprised of an Exposure and a Test phase. During Exposure, 21 non-musicians were instructed to detect a timbre shift (TS) within short microtonal melodies, and we hypothesised that they would incidentally learn about the pitch interval structure of the microtonal scale from attending to the melodies during the task. In a follow-up Test phase, the tone before the TS was either a member (congruent) or a non-member (incongruent) of the scale. Based on our statistical manipulation of the stimuli, incongruent tones would be a better predictor of an incoming TS than the congruent tones. We therefore expect a faster response time to the shift after the participants have heard an incongruent tone. Specifically, a faster response time observed after an incongruent tone would imply participants’ ability to differentiate tones from the microtonal and the diatonic scale, and reflect their learning of the microtonal pitch intervals. Results are consistent with our predictions. In investigating the learning of a microtonal scale, our study can offer directions for future research on the perception of computer music and new musical genres.

## Introduction

Music has become more accessible since the late 20th century when different music genres and world music from all over the globe can be freely obtained through the media. Creating music can be achieved by using computer software, which opens room for non-traditional music. Past research has provided insights on how humans perceive different attributes of Western music, including tonality, rhythm and timbre [1–3], and how enculturation shapes our music perception [4,5]. However, the accessibility and diversity of music creation nowadays make it sensible to expand our research on understanding how one approaches novel musical styles and structures. To achieve that, collecting baseline data of people learning a new music system is essential. In this paper, we examined the learning of a musical scale that is novel to listeners of all backgrounds. It is a computer-generated musical scale which shares only limited features with Western music, and is unlike most music from other cultures [6].

Discussions on how we became familiar with a music system through enculturation [7–9] or within an experimental session are longstanding. Past research had suggested that we learn from an early age about the compound structure of our native music [10–12]. We also have the ability to statistically learn unfamiliar musical structures including finite state grammars [13,14], musical scales from another culture [15,16], or sequences of musical events [17,18]. These conclusions were inferred from data on goodness of fit ratings [19–21] or forced-choice responses on level of familiarity [22,23]. In other words, if the participants had considered certain stimuli as fitting better or being more familiar, the authors would assume the participants had learnt that those stimuli followed the artificial grammar or musical structure. The findings were consistent, yet limited by the range of target stimuli being tested as they were usually Western tonal music or music from a limited range of cultures. In order to obtain a better understanding of how fast and how well listeners could learn a novel music system, we examined the incidental learning of a microtonal scale that is unfamiliar to listeners from any cultural background.

Incidental learning is defined as one type of statistical learning, where participants are exposed to structured materials without being told to learn or analyse them in relation to the parameter to be measured [24]. In the current study, participants were not expected to be conscious of the statistical regularities of the materials that we manipulated based on the newly designed paradigm. We argue that this paradigm is a more sensitive measure of statistical learning than the past paradigms. While we expect participants will be unaware of the statistical manipulation of the paradigm, their ability to detect pitch intervals that are incongruent with those of the novel scale (violating the pitch structure of the scale) is inferred by their reaction time (RT) to a timbre shift (TS). The current paradigm can be used not just for measuring the learning of musical structures but also stimuli in other modalities.

### Diatonic vs microtonal scales

A music system may consist of instrumental musical sounds, environmental sounds, and other digital sounds, and could be characterised by its tone quality (timbre), pitch range and intervals, intensity, and so on. Western classical music is one kind of music system, with specific sets of pitches, a single adjacent pitch interval ratio, and predominantly based on acoustic instruments and electronic counterparts that mimic them and that rely on the same pitch structure. In this paper, we focus on musical pitch systems, which include tuning systems and musical scales. A tuning system is comprised of a selected group of pitches, which are describers of musical tones in the system. For example, 12-tone equal temperament (12-tet) is the common tuning system used in Western classical music, where one octave is logarithmically divided into 12 equal steps, which forms a fixed interval ratio between pitches in the system. Tonal hierarchies could be perceived amongst pitch relationships in a tuning system, where for example two notes an octave (12 semitones) apart are perceived as better fitting than when one is two semitones away. Tonal hierarchy in a musical context involves this idea that certain musical tones are more stable and structurally significant than others in that context. For Western musical scales, the first tone of the scale (the tonic) is perceived as the most stable tone, which heads the hierarchy. It is followed by the fifth and third scale degrees (the dominant and the mediant, respectively), the other (major or minor) scale tones, and finally the non-scale tones [2,25]. Past research has shown that non-musicians are highly sensitive to the tonal hierarchy in Western music [26].

A Western musical/diatonic scale (e.g. C major scale) is a subset of seven pitches from the 12-tet system, and it consists of a pattern of small and large step sizes (a small step in a diatonic scale equals one semitone, while a large step equals two semitones or one tone). To examine the learning of a musical scale, it is preferable to use a novel stimulus that listeners have never been exposed to (see review paper [27]), and an algorithmically formed microtonal system is a perfect tool for this purpose. Microtonal systems are commonly defined as a music system with pitch intervals smaller than a semitone, but this definition has been extended to any pitch intervals that are different from 12-tet. For instance, 11-tet is a microtonal scale that divides an octave into 11 equal steps, while the 81-primes scale [28] has unequal step sizes throughout without octave divisions and additive rather than the normal multiplicative pitch differences between members, and is defined based on prime numbers. The current study has chosen a microtonal scale that has two features similar to the diatonic scale to facilitate learning: being “well-formed” [29] and having an asymmetrical pattern of small and large step sizes.

**Fig 1:**
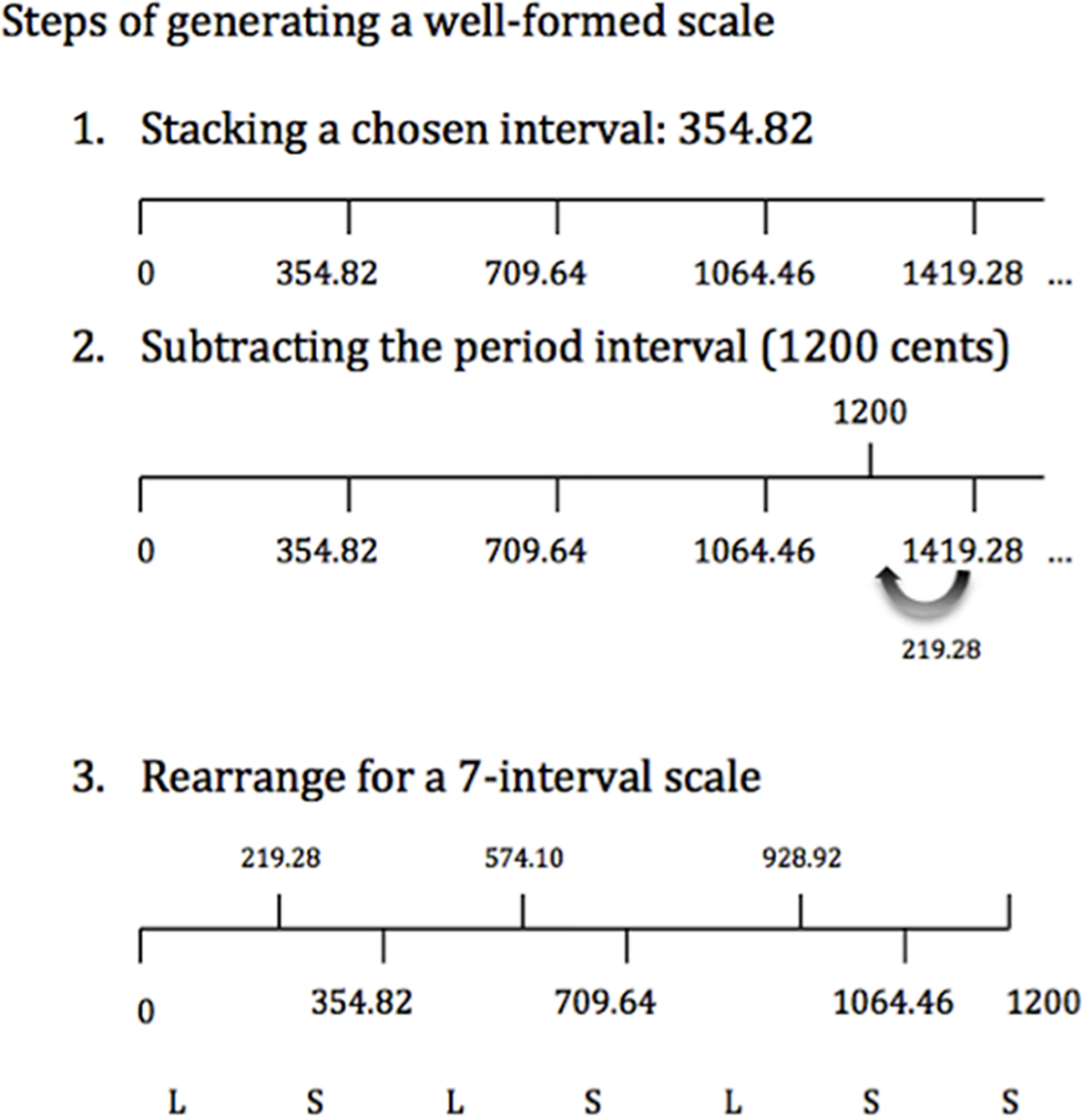
The steps of generating a well-formed microtonal scale with a generative interval of 354.82 cents. The Ls refer to large steps and the Ss refer to small steps in the scale. The intervals of the large step and the small step are 219 cents and 136 cents (rounded up) respectively.

A well-formed scale is a structured division of an interval of repetition and it contains two evenly distributed step sizes. It is created by stacking a generator interval (usually a perfect fifth) repeatedly within the boundary of a particular period (typically an octave). When the interval size exceeds the period, the period interval will be subtracted. With the repetition of interval stacking and period interval subtraction, different step sizes are formed and they will be evenly distributed if the scale is well-formed (see Fig 1). The number of large steps and small steps in each period is always co-prime (no common factors besides 1), which makes the scale asymmetrical [30]. In our experiment, a microtonal well-formed scale is created by using the generator interval of 354.82 cents, but it follows the same rules and has four small and three large steps. We only use one octave in this experiment, with the first tone starting from 262Hz (pitch frequency of the Middle C / C4 in 12-tet). Due to their relative pitches being different from 12-tet, we do not expect Western listeners to be familiar with them. Indeed, an algorithmically developed microtonal system allows us to manipulate the pitch interval ratios, the number of pitches/steps over a period (described as an octave in the diatonic scale), the event frequencies of individual pitches, pitch range of the period, and so on. The possibility of manipulation is close to unlimited but what we are interested in here is: 1. Can listeners learn the pitch intervals of a microtonal scale through mere listening to the melodies created from this scale within an experimental session; 2. Can listeners retain their knowledge of the new scale in a longer term?

### The possibility to learn such a novel scale

In our experiment, learning a microtonal scale involves learning the pitch intervals created by the relative pitches of the set of tones that are used to generate the melodies, our stimuli. Since we can learn about the pitch intervals of our familiar music system through prior exposure, we assume being exposed to those microtonal melodies would elicit the same form of learning. Such learning can be reflected by one’s ability to discriminate items that are congruent with our knowledge of the system and those that are not. According to previous literature on musical expectations [31], an out-of-key tone embedded in a melody would create a ‘surprise’. This surprise or rise of awareness/alertness is evoked by the listeners’ ability to discriminate between tones that create the ‘right’ pitch intervals (that belong to the scale of the melody) and those from a different scale. Other studies have also evidenced that we can efficiently detect incongruent musical events, as long as we know the musical structure of the presented music well enough. This is not just the case with music that we are acculturated with [32,33] but also with newly acquired musical knowledge during experimental sessions. Saffran etal [18] found that adult and 8-month-old participants who were exposed to a repetition of ‘tone-words’, which were musical tones with defined syntactic patterns like syllables in words, showed better-than-chance identification performance in an immediate test on indicating which tone-word in the presented pair was more familiar to them. It was suggested that participants were able to track the induced probability of the co-occurrence of particular pitches that formed the tone-words as well as the pitches themselves. The consequence of this learning had led the participants to reject items that were incongruent with those probabilistic patterns in the immediate identification test. Along similar lines, examinations of artificial grammar learning of an unfamiliar musical scale (Bohlen-Pierce scale in [16]), or of musical pitch or timbre sequences [14,34,35] were conducted by observing participants’ reaction time or familiarity response to congruent and incongruent items. Incongruent or less related items were consistently rejected or responded to slower than congruent items, which followed the to-be-learnt grammar or musical structure. Lengthened reaction time was suggested to be due to the awareness of incongruent items, which had led to a longer processing time during the required response. This difference in reaction time would only be observed if those items were discriminated from the congruent ones. Therefore, testing the awareness of incongruences is an effective way of measuring one’s familiarity to the materials, and this has formed the basis of our paradigm.

### Our paradigm

We envisaged the following paradigm for examining the incidental learning of a microtonal scale during a timbre shift (TS) detection task. This task involves detecting a TS (from piano to clavichord) in one of the notes within a short melody. The unusual feature of this paradigm is its manipulation of the relative frequency of the type of note (being a member of the local or an alternative musical scale to the melody) immediately before a TS, such that an incongruent tone has an increasing likelihood to be followed by a TS. (Most prior paradigms manipulate only features of the tone to which a response is required, and often conflate several simultaneous manipulations in so doing, thus complicating interpretation). The increasing likelihood will make incongruent tones a good predictor (valid cue) of the shift, and therefore affect participants’ RT in responding.

**Fig 2:**
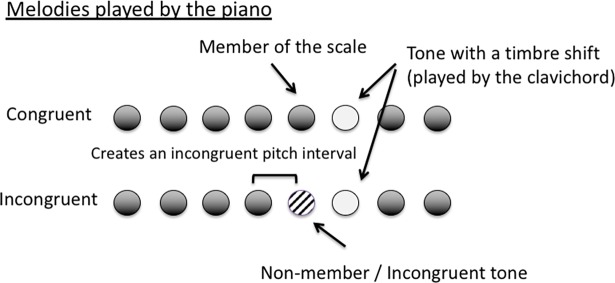
In an experimental trial, a melody played in piano timbre is presented. In the congruent condition, all the tones are members of the musical scale while in the incongruent condition, there is a non-member/incongruent tone embedded in the melody. When there is an incongruent tone, it creates an incongruent pitch interval with the tone before it. Participants’ task was to detect and respond to the tone that has a TS as quickly as possible.

In a typical trial, participants are presented with a short 8-tone melody generated in synthesised piano timbre. Depending on the testing condition, most/all of the tones are members of one musical scale (local scale of the melody). Any incongruent tone (a non-member of the scale) embedded in a melody will create an incongruent pitch interval with the note before it (see Fig 2). The idea is to test whether listeners can detect that incongruent interval. To better reflect that ability, we manipulated the probability of an incongruent tone preceding a TS to increase in Test phases of the experiment, so that it will progressively be learned to be a good predictor of the shift. If participants respond faster to the TS after hearing an incongruent tone, we can deduce that they have perceived that tone as being a non-member and had a high expectation of an incoming TS, as there is a relatively high positive statistical relationship between these two events. However, this response pattern will only be observed if the participants have become familiar with the pitch intervals of the microtonal scale and have learnt the statistical association between the incongruent tone and the TS during the exposure and the test phases. If they had not sufficiently learned the two, they would not be able to differentiate between the congruent and the incongruent intervals, and their RT to the TS would be similar to that in conditions without any incongruent pitches.

While this approach seems novel in experimental music research, the current paradigm is analogous to the masked priming paradigm in multisensory research (manipulating the event before the response target, or the prime, which affects the response time to the target that comes after, see [36,37]). The effect of priming with congruency manipulation is also commonly used in psycholinguistics research such as semantic priming in a lexical decision task (e.g. [38,39]). In such a task, participants are presented with the prime followed by a target, then required to make a decision, such as whether the target is a word or a non-word. Speeded responses to the target were observed when the prime was semantically related to the target. Posner etal [40] argued that manipulating the probability of a particular prime preceding a response target activates an expectancy of such a relationship and the presence of that prime becomes a cue to the target. With the cost and benefit analysis they proposed, it is more cost-effective in terms of cognitive demand for the participants to form such expectations, and therefore it takes longer for them to respond to the target when it has not been cued by the right prime. In our paradigm, the ‘right’ prime would be an incongruent tone as it is likely to be followed by the response target, which is a TS. Once participants have learnt the association between an incongruent tone and the TS, the presence of an incongruent tone becomes a cue to a highly possible timbre change. Response time to the TS will therefore become faster. However, if a TS occurs when the cue is absent (only hearing the congruent tones), we would observe a lengthened RT to the shift due to its unexpectedness.

Furthermore, unlike previous studies that manipulated both the congruency and timbre shift on the response target itself [14,41], this paradigm dissociates the response target (timbre shift) from the pitch shift that creates the incongruence. This will avoid the possible influence of timbre on the perceived pitch [42] of the items, which is crucial in this study. With the pitch shift happening before the timbre change, we can be certain that any difference in responding to the target would be a direct influence of the incongruent intervals created by the pitch shift. Besides, with our paradigm the response target does not necessarily have to be in the form of a TS. It can well be a beep or a burst of white noise. Keeping it as a pitched timbre change allows us to check if the pitch range of the response target would be an external factor on reaction time, which previously has been neglected in this type of testing paradigm.

### Hypotheses

We hypothesized that familiarity with a musical scale would allow participants to become sensitive to incongruent pitch intervals. Therefore, if participants have sufficiently learnt the pitch intervals of the microtonal scale during the exposure phase, we expect them to perceive pitch intervals created by pitches from a different (diatonic) scale as incongruent. Similarly, we assume the participants will also be sensitive to deviant pitch intervals appearing in the diatonic melodies. The ability to detect incongruent pitch intervals will be implied by their learning of the statistical association between the incongruent tones and the TS. If participants have learnt that statistical relationship, we will observe a speeded response to the shifts in the incongruent condition. This response pattern would suggest that participants are able to perceive the difference between congruent and incongruent pitch intervals, and a less expected congruent-TS pairing would lengthen RT.

## Materials and methods

This research was conducted according to the principles expressed in the Declaration of Helsinki and approved by Western Sydney University’s Human Research Ethics Committee (approval number: H10669). All participants provided written informed consent.

### Design

The experiment is comprised of two sessions that are spaced one-week apart. The first session involves performing an Exposure and an Immediate Test for each musical scale condition. The session a week later requires participants to perform a Later Test, which aims to measure long-term memory of the learnt system from session 1. There are three independent variables (IVs): 1. Scale (C Diatonic/Control vs Microtonal); 2. Test Phase (Immediate vs Later); and 3. Congruency (Congruent vs Incongruent). The dependent variable is the RT in correct detection. In both the Exposure and the two Test phases, the format of the timbre shift detection task remains the same, but the stimuli differ in terms of the musical scale and congruency conditions.

**Fig 3:**
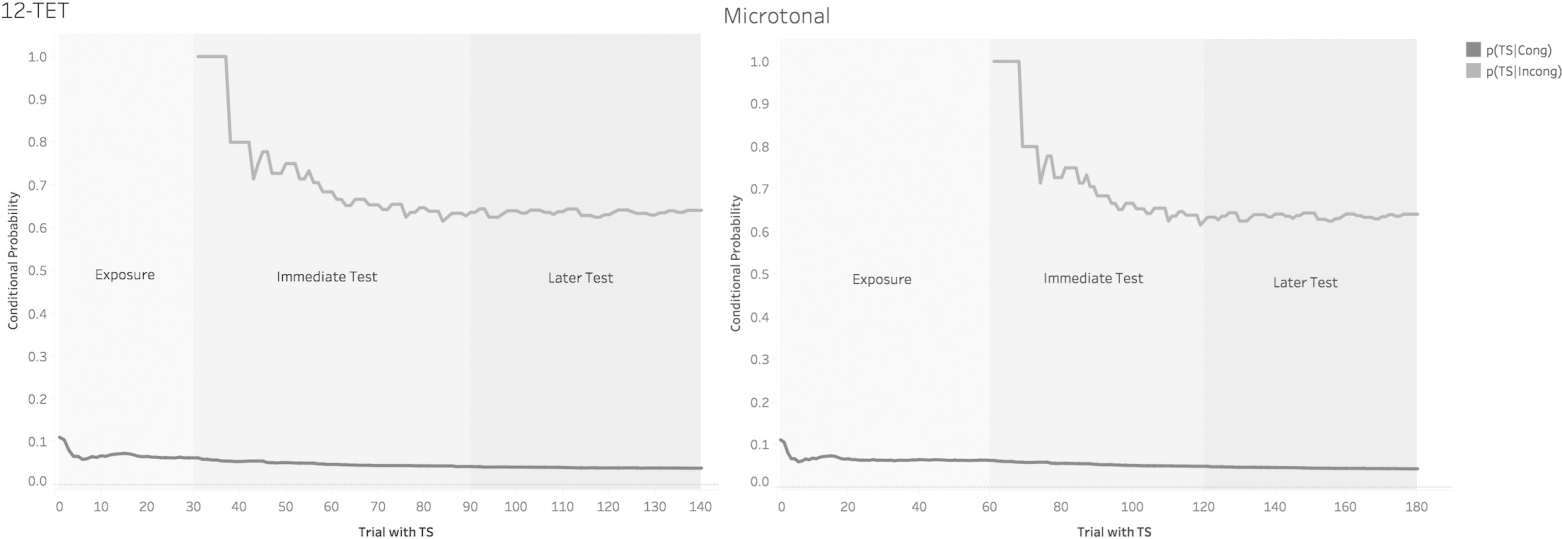
The conditional probability (by trial, across participants) of a congruent or an incongruent tone being followed by a TS in the exposure and test phases.

In the exposure phase, tones in the melodies are generated from one musical scale only (Control or Microtonal), and there is a timbre shift in half of the trials. In other words, incongruent tones are not presented during the exposure phase. In the Immediate and Later test phases, the tone before the TS can either be congruent (members of the local scale (scale of the melody)) or incongruent (members of another scale with the closest pitch to the original tone, that not conforming with the ongoing melodies). The hypothesis that RT will be shorter in the Incongruent condition is made based on the conditional probability that TS occurs immediately after a congruent (abbreviated as p(TS|Cong)) or an incongruent tone (abbreviated similarly as p(TS|Incong)). In the exposure phase, there is an equal chance of a timbre shift appearing or not appearing after a congruent tone due to the equal number of trials that include or do not include a timbre shift. During the Exposure phase, the average p(TS|Cong) therefore equals the total number of times a TS occurs after a congruent tone/the total number of congruent tones presented in the Exposure, i.e. 07, which is very low. When participants begin the Immediate test, they are expected to have learned that they cannot rely much on congruent tones to predict an incoming TS. However, when participants start hearing the incongruent tones, the p(TS|Cong) gradually reduces even further. That is because a timbre shift can now be preceded by an incongruent tone. In fact, most of the time, an incongruent tone will be followed a timbre shift, such that the average p(TS|Incong) will be .63 by the end of the Immediate test and remain similar in the Later test (see Fig 3). Fig 3 represents these two conditional probabilities, by trial number but averaged across all participants. Thus p(TS|Cong) is constant at .07 during Exposure, and falls gradually thereafter; while p(TS|incong) is zero during the exposure phase, but 0.63 throughout Immediate and Late Test phases.

### Stimuli and materials

Stimuli are 8-tone melodies generated from either the C major (Control) or the microtonal scale, with a note duration of 400ms. Three sets of melody index are generated using an algorithm written by YL in the Max software (Cycling ‘74); one for the exposure phase and one each for the Immediate test and the Later test. Each melody is generated by choosing the first note randomly from the scale, and the successive notes are based on the number of semitones/steps from the note before it. In order to avoid repetitions of notes within a melody, a simple random sampling without replacement is conducted in the selection of the successive notes (all the potential notes have an equal chance of being selected, and the ones that have been selected previously are excluded from the pool). Tones in melodies between Control and Microtonal conditions are matched in terms of note arrangement of members of the scales. Pitch adjustment of the microtonal melodies was performed using the HexBridge Max Patcher written by Andrew J. Milne. The pitch range of the microtonal melodies is equivalent to two octaves in the C major scale (262 to 1048Hz). A C major scale is a selection of seven pitches from the 12tet system, with a consistent pattern of two small (1 semitone) and five large (2 semitones) step sizes across octaves/periods (1 semitone = 100cents). The microtonal scale used here partially resembles it. It has seven pitches in each period with two step-sizes (small: 136 cents; large: 219 cents). However, there are three large steps and four small steps in each period. The beginning and ending pitches in each period are the same as the diatonic scale, but the pitches in between are different. While the diatonic scale has a pattern of L (Large step), L, S (Small step), L, L, L, S; the microtonal scale has a pattern of L, S, L, S, L, S, S. The interval between the current and the next note is controlled, such that the largest possible pitch interval between notes is seven semitones/small steps and a large interval will usually be followed by a smaller interval (based on the tendency of pitch intervals in 12-tet music suggested by Narmour’s implication-realization model [6]). We do not assume that listeners will approach the new scale the same way as the diatonic scale.

Here is how a melody is generated in full. Using a diatonic melody as an example, after the first note of the melody has been chosen from a diatonic scale, each successive note is randomly selected from the pool of notes that are no more than seven semitones apart from the current note using the method of simple random sampling. Because of that, there is no repetition of notes within each melody. Seven-semitones is equivalent to seven small steps or 3 large steps (1 large step = 2 small steps) and 1 small step in the diatonic scale. When a melody is generated from the microtonal scale, the generation of successive notes will follow the same rule: equal or less than seven small step sizes. The successive note is always generated with reference to the note before it. In other words, the third note will be generated based on the pitch of the second note (no more than seven small steps from the second note), and the fourth note will be chosen based on the third note. Since the maximum interval size between notes is seven semitones/small steps, large intervals will often be followed by the same or a smaller interval due to the higher probability of small intervals. For notes that are closer to each end of the two-octaves range, the number of possible successive tones will reduce. The contour of the melodies is not controlled, and the successive notes can have a pitch higher or lower than the current note. There is no repetition of notes in the melody, thus the pool of available notes at any point excludes those that have been sounded already.

The timbre of the melody and the tone with a TS are synthesised with the physical models installed in Pianoteq 5 Pro (version 5.1.4/20150211), which means that any pitch can be synthesised without interpolation (and audio samples are not involved). The melodies are played in D4 Daily Practice (modified), which resembles the timbre of a grand piano. The note with a timbre change is played in Neupert Clavichord single, which resembles the timbre of a clavichord. Attack time (time of peak amplitude) measured by Audacity was 0.011s for the piano timbre and 0.013 for the clavichord. Stimuli and responses are presented and recorded using Max (presentation and interface are scripted by YL), Pianoteq 5 Pro, and AU Lab (v.2.2.2) using a MacBook Pro laptop. Melodies are played through a pair of headphones (Sennheiser HD 280 Professional) and participants completed two questionnaires about their musical background, the Ollen Music Sophistication Index [43] and the Goldsmith Musical Sophistication Index (Gold-MSI) [44].

### Trial arrangement

There are 120 trials in each test phase and in the Microtonal exposure phase, but only 60 trials in the exposure phase of the Control scale, to shorten the testing time. It is assumed that participants have already had extensive exposure to the Control scale so that half of the exposure time compared to the microtonal scale would be sufficient. Consistent with this, starting the session with the Control condition will resemble real-world situations when listeners first experience a microtonal scale. Reversing the order might lead to a carryover effect of the microtonal condition on their perception of diatonic melodies immediately thereafter. The tone with the TS is randomly located from the 3rd to the 7th note, while the incongruent tone is normally located immediately before it. There are no TS in the first, second or the last note of the melody. In a small number of trials, an incongruent tone is randomly located at any other position before or after the note with altered timbre. While these trials can be used as catch trials, any false alarms (responding immediately after the incongruent tone) would likely indicate a participant’s sensitivity to the incongruent tone. There are also a small number of trials with no TS, but with an incongruent tone embedded in the melody and false alarms immediately after these tones can be interpreted similarly (see Table 1).

**Table 1:** Number of trials per condition. In the analyses, congruent and incongruent conditions refer to the trials in the immediate and later tests and that has a TS. We are expecting possible false alarms in trials with no TS but consist of a randomly located incongruent tone.

Session	Experimental phase	Scale of the melody	No TS		TS		
			[Congruent] All tones from the same scale	[Incongruent] With a randomly positioned tone from another scale	[Congruent] All tones from the same scale	[Incongruent] Tone before TS is from another scale	[Manipulation check] A randomly located incongruent tone (not presented immediately before the TS)
1	Exposure	Control	30	0	30	0	0
	Immediate test		50	10	30	25	5
	Exposure	Microtonal	60	0	60	0	0
	Immediate test		50	10	30	25	5
2	Later test	Microtonal	50	10	30	25	5
	Later test	Control	50	10	30	25	5

### Participants

21 non-musicians recruited from June to July, 2015 from Sydney, Australia through the MARCS Institute, Western Sydney University website completed both sessions of the experiment (Mean age = 30.2 years, SD = 5.79 years). While the sample size was not determined by power analysis, individual differences were examined and controlled by the mixed effects model conducted on all the collected data. All participants have less than five years of formal musical training (both instrumental and voice), and their mean OMSI [43] score is 91.65, (respondents with a score higher than 500 are classified as being more musically sophisticated). More detailed musical background information was collected using the Gold-MSI [44], which is considered as a more sensitive measurement of musical sophistication of the general population, while OMSI was developed based on professional musicians and with a stronger emphasis on classical instrumental training. The Gold-MSI assesses skills that could apply to a wider range of musical practice (both classical and contemporary) and it considers self-assessed musical skills. Five Gold-MSI subscales were used for measuring factors that would significantly predict musical sophistication:

1. Active Engagement (e.g. “I spend a lot of my free time doing music-related activities”)
2. Perceptual Abilities (e.g. “I am able to judge whether someone is a good singer or not”)
3. Musical Training (e.g. “I engaged in regular, daily practice of a musical instrument (including voice) for __ years”)
4. Singing Abilities (e.g. “I can sing or play music from memory”)
5. Emotions (e.g. “I sometimes choose music that can trigger shivers down my spines”)

By incorporating the aspects of all five factors above, the index provides a score of the General Music Sophistication factor together with sub-scores of each of the five subscales (see S1 Table).

### Procedure

In the first session, participants started with the exposure phase in the Control scale (diatonic scale), followed by an Immediate test. In the Incongruent condition of the test, one of the notes in the melody was generated from the Microtonal scale. After that, the exposure phase of the Microtonal scale began, and then an Immediate test phase followed where the incongruent tone was generated from the Control scale. Participants then came back a week later for a second session. They performed the second/Later test phase in the Microtonal scale, which allowed testing long-term learning from the exposure in the first session. They finished the experiment by performing the Later test of the Control scale and completing questionnaires about their demographic information and musical background.

### Validation of the design

Reaction times in the Exposure phase are considered as a baseline measure of response time to the timbre change in a condition where no deviant tone was present. They were compared with the reaction time in the Congruent condition in the Immediate test as a manipulation check. If they did not differ significantly, it would suggest their reaction time to the timbre shift in the Congruent condition is consistent and any differences in reaction time between the Congruent and Incongruent conditions are due to our manipulation. A Repeated-measures Analysis of Variance (ANOVA) that compared reaction time between Exposure and Immediate test-Congruent in both Scales found no significant difference, Control: *F*(20) = .33, *p* = .58; Microtonal: *F*(20) = .54, *p* = .47, which validated our design.

## Results

### Statistical approaches

Data are analysed using linear mixed-effects models (see S1 Text) for basic principles of these models. Basically, we used mixed effects models to examine the possible factors of timbre shift detection ability, including pitch sensitivity and stimulus-related properties such as pitch distance between the original and the incongruent pitch intervals in each trial of the Incongruent condition. More importantly, these models allow us to examine the trajectory of performance change over the time course (measured by trial) of the task, which indicates whether the statistical learning of the stimuli is successful. This cannot be achieved by conducting univariate tests such as ANOVAs.

All analyses were conducted using RStudio (v. 0.98.1087), with the R packages ‘lme4’ for the mixed effects analysis with function ‘confint’ to calculate the 95% confidence intervals of the fixed effects (factors of the performance). The models will analyse the performance based on: 1) the reaction time in correct timbre shift detections; 2) the hit rate in detecting a timbre shift, and 3) the false alarm rate in trials where there was a randomly located incongruent tone but no timbre shift. Responses with reaction time less than 200ms or longer than 1500ms after the onset of the tone with timbre change were considered as incorrect and were rejected from the analyses.

### Reaction time in correct timbre shift detection

**Fig 4:**
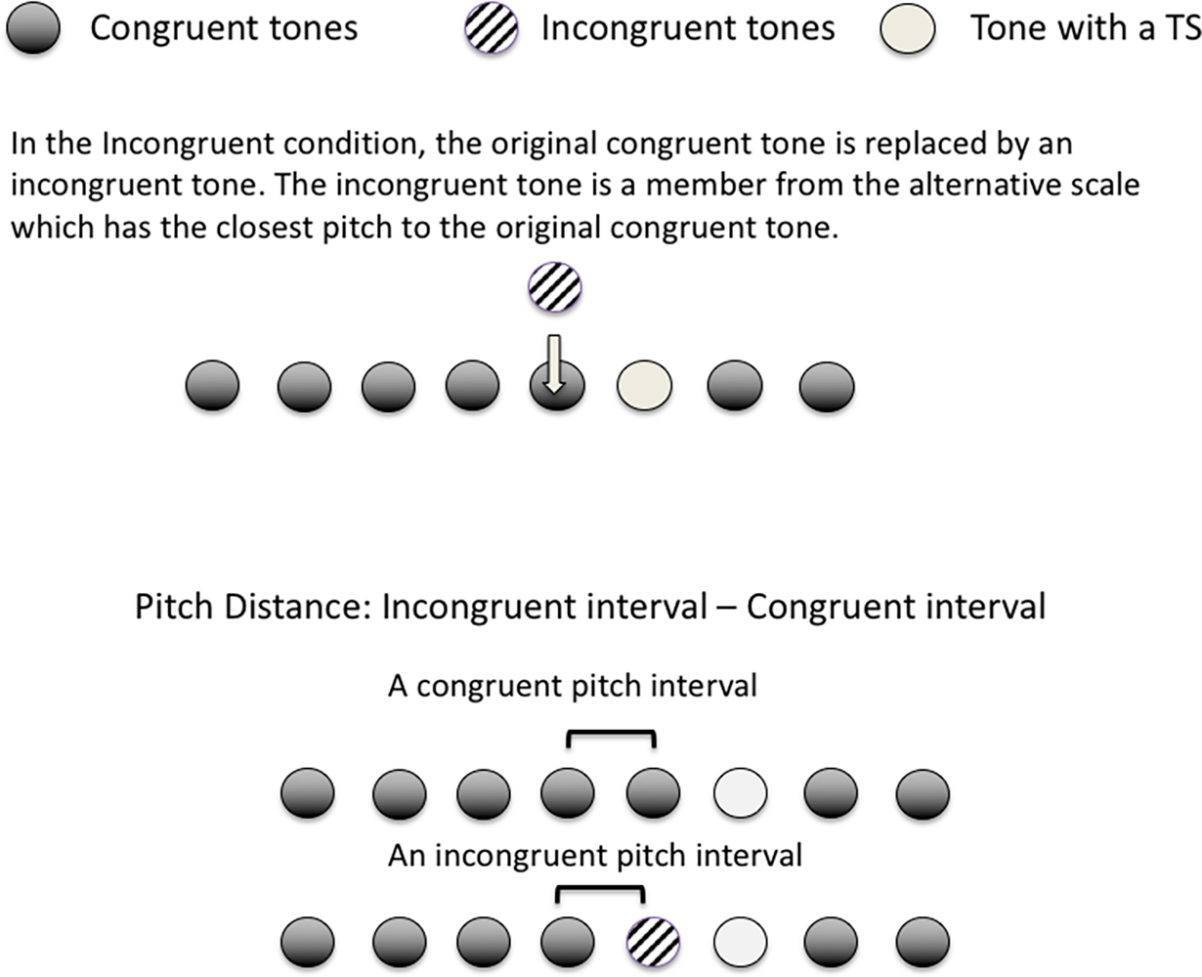
The definition of pitch distance (fixed factor 4), which is the absolute difference between the congruent and the incongruent pitch interval between the tone before the TS and the one before it.

Linear mixed effects models were conducted on the reaction time of correct timbre shift detection in the Immediate and Later tests. Fixed factors considered include:

1. Test Phase (Immediate vs Later). If participants have retained the knowledge of the microtonal scale from the initial exposure, we should see a similar response pattern in the Later test.
2. Congruency (Congruent vs Incongruent conditions). Faster response time in the Incongruent condition is expected.
3. Scale (Control vs Microtonal). Using responses to the Control scale as our baseline data, a similar response pattern to the Microtonal scale would imply learning.
4. Pitch Distance between the incongruent and the corresponding congruent (the closest note in the parent scale of the melody to the incongruent note) intervals. In other words, the difference in pitch interval an incongruent tone has made (see Fig 4). The larger the difference, the more noticeable the incongruent tone is.
5. Position of the tone before the timbre shift.
6. Time/Trial number, which reflects the timeline of the experiment. The bigger the number, the closer it is to the end of the experiment. Treating this as one of the fixed factors allows us to check if reaction time becomes shorter over time due to the changes in p(TS|Cong) and p(TS|Incong), which implies learning. Values of the Trial number have been scaled by mean centering, where the mean of the total trial numbers is subtracted by each trial number, and the resulting value forms each value of the variable.

**Fig 5:**
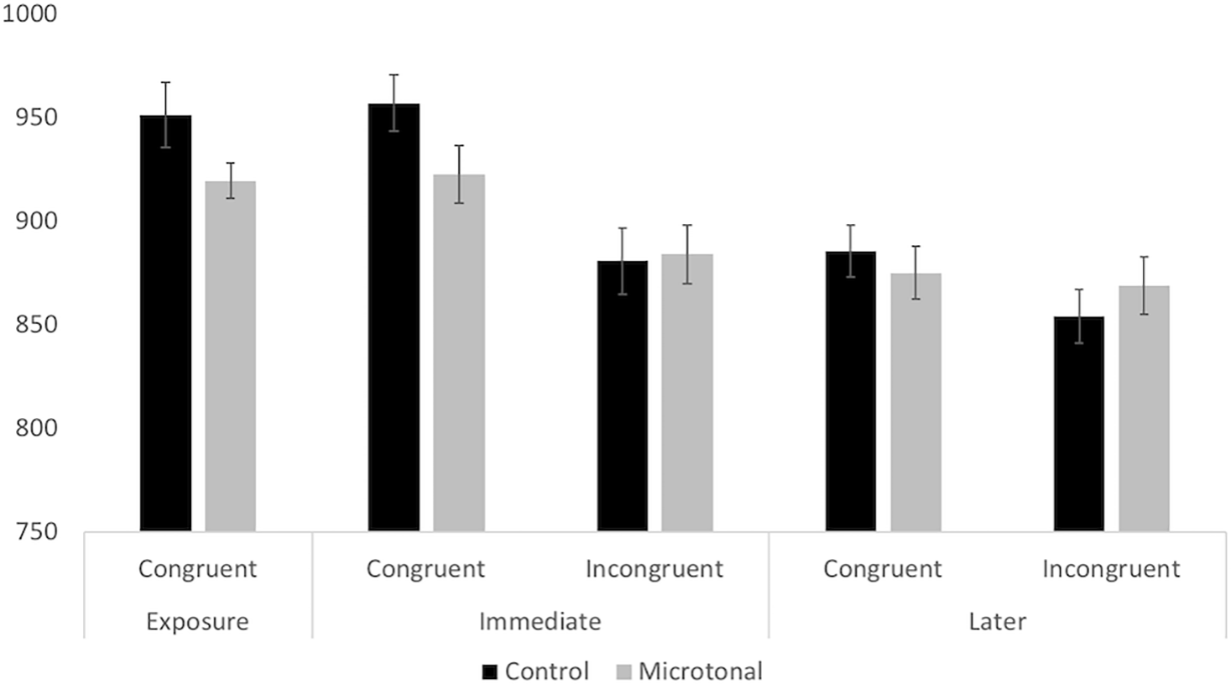
RT (in millisecond) between test conditions. Error bars show 95% confidence intervals (throughout).

The approach here is to first analyse the whole dataset to compare reaction time between musical scales, congruency, and test phases. Afterwards, we analyse data from the congruent and the incongruent conditions separately. This is sensible because fixed effects such as pitch interval difference (4. Pitch Distance) are not relevant to data in the congruent condition. The models consider individual differences as a random factor. They include the maximal random effects driven by the experimental design, an approach suggested by Barr etal [45]: in this case, the assumption that the intercept differs between participants. Results reported below are the models that best predict the data after model comparisons using the Chi-square likelihood ratio test. Fig 5 shows the mean reaction time of the correct timbre shift detections across Test phases, Congruency and Scale conditions. The overall hit rate was above .89. In the Immediate Test phase RTs were 39–76 msec shorter in the incongruent than in the congruent conditions; and this difference decreased in the Later phase for the familiar Control scale conditions and lost for the unfamiliar Microtonal conditions.

**Fig 6:**
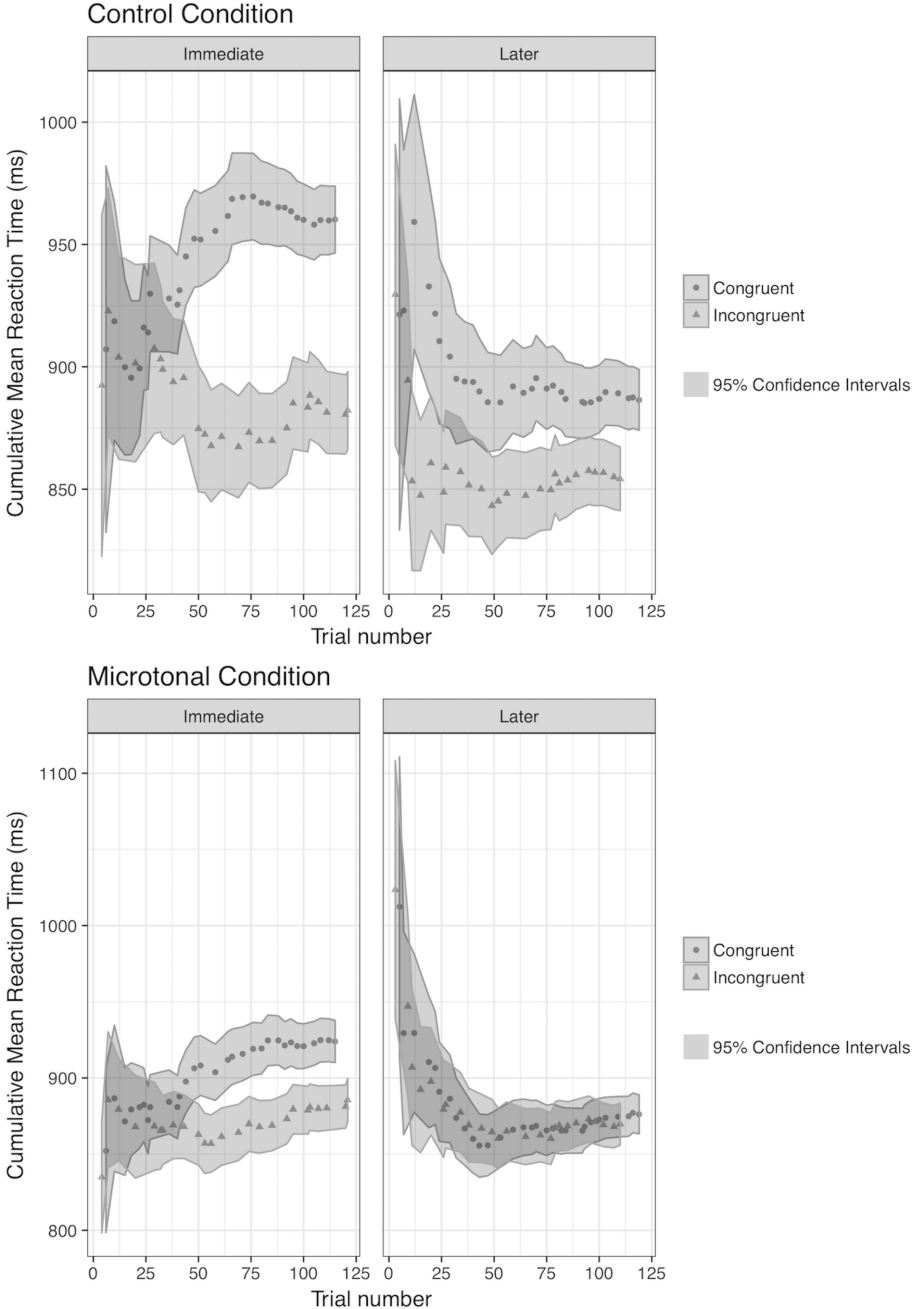
Cumulative mean reaction time in correct detection of timbre shift among non-musicians in the control and the microtonal conditions of the test phases.

In addition, Fig 6 shows the cumulative mean reaction time over test phases in each Scale condition. Cumulative mean here is a progressive average of reaction time over individual trials and each successive dot represents the mean reaction time up to and including the trial where the dot is located. The 95 percent confidence intervals calculated from the cumulative standard deviations of the mean are shown in the shaded area of the graph. It gives a meaningful representation of the change in reaction time over trials. A gradual and stable decrease in reaction time in the Incongruent condition would provide a strong evidence that learning has taken place. As predicted, the graphs support and extend the bar graphs in Fig 5. Fig 6 shows the predicted progressive changes in the Immediate test phase, but also that in the Later test phase. There is a progressive improvement (shortening) of reaction time down to levels that are if anything better than in the Immediate test phase, and such that the difference between Congruent and Incongruent in the Microtonal condition is lost within the first 30 or so trials.

We will first present the results from the mixed effects models for the RT data. The first model analysed the RT data from all conditions. The second and the third analysed the RT in the Congruent and the Incongruent conditions respectively.

#### RT across congruency conditions: Model 1

In Model 1, the influence from fixed effects of Congruency, Scales, Test Phases, Trial, and the Position of the tone before the timbre shift on RT was analysed. We also assumed a possible interaction between Scale and Congruency. There is only one random effect, which is the response of a participant to the progression of trials. Results confirm that RT decreases significantly in the Microtonal condition and in the Later test. It was significantly shorter in the Incongruent condition and when the timbre shift happened later in the melody. There is a significant interaction between Scale and Congruency, such that the RT between congruency conditions varies upon the scale of the melody. As shown in Fig 6, the RT difference between Congruent and Incongruent conditions is larger in the Control condition compared to the Microtonal condition. The random effects on Trial by Individuals were considered: the assumption that RT changes over trials and the change depends on individual participants was considered in the model. However, it will be more sensible to discuss this in the next two models when RT data is separated between congruency conditions.

#### RT for congruent trials: Model 2

In Model 2, only correct RT data from the Congruent condition was analysed, as fixed effects relevant are different from those in the Incongruent condition. Fixed effects include Trial, Tone Position and the interaction between Scale and Test Phase. We assumed RT would increase over congruent trials due to the decreasing p(TS|Cong) and vary among participants (random effect). As predicted, RT increased significantly over time (fixed effect of Trial). Comparing between Scales and Test Phases, RT was significantly shorter in the Microtonal condition and in the Later test. Tone Position was significant, such that participants responded faster to the TS when it appeared later in the melody. The interaction between Scale and Test Phase was significant, that participants responded faster to the TS in diatonic melodies in the Later (than the Immediate) Test but similarly to those in the microtonal melodies.

#### RT for incongruent trials: Model 3

**Fig 7:**
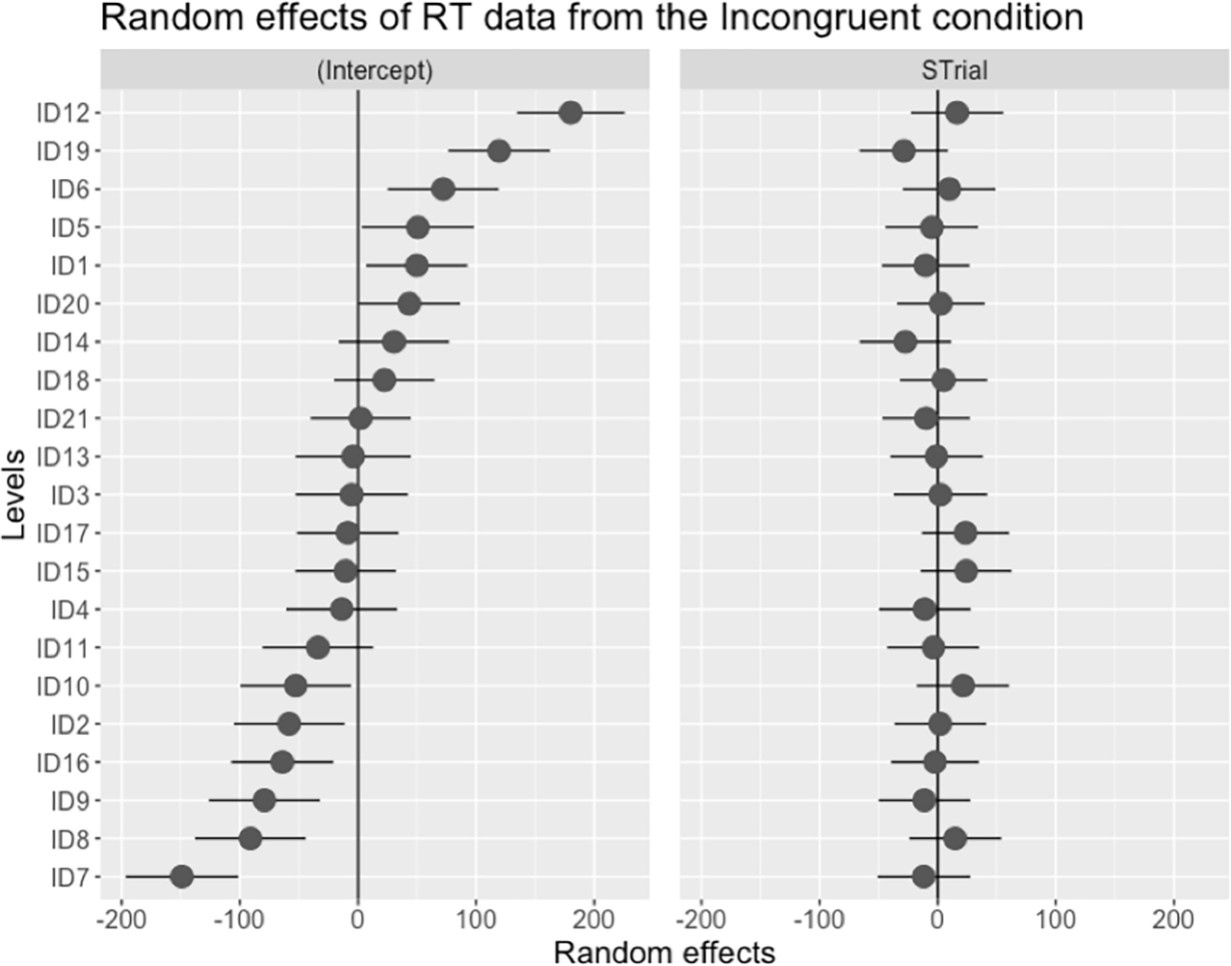
This diagram shows the intercept values and the random coefficients of reaction time of each participant across trials in the incongruent condition. ‘STrial’ stands for the scaled value of trial number using the centering method.

The third model analysed reaction time data from the Incongruent condition, with an additional fixed effect of Pitch Distance (which was not relevant in the congruent condition). It was predicted that the bigger the difference between the original congruent and the replaced incongruent pitch intervals (see Fig 4), the faster the RT to the timbre shift. Results indicated that fixed factors of Test Phase and Tone Position were significant. RT was significantly shorter in the Later Test and when the TS appeared later in the melody. RT over trials varied mildly between participants, as shown in the coefficients of the random effects (Fig 7). Unlike the RT in the congruent trials, RT did not change significantly over the time course of the test, perhaps due to the relatively high and hardly changing p(TS|Incong). In other words, once the incongruent tone was a better predictor of the timbre shift than the congruent, the reaction time increased in the Congruent condition, but was relatively stable throughout in the Incongruent condition.

### Hit rates for timbre shift detection

**Fig 8:**
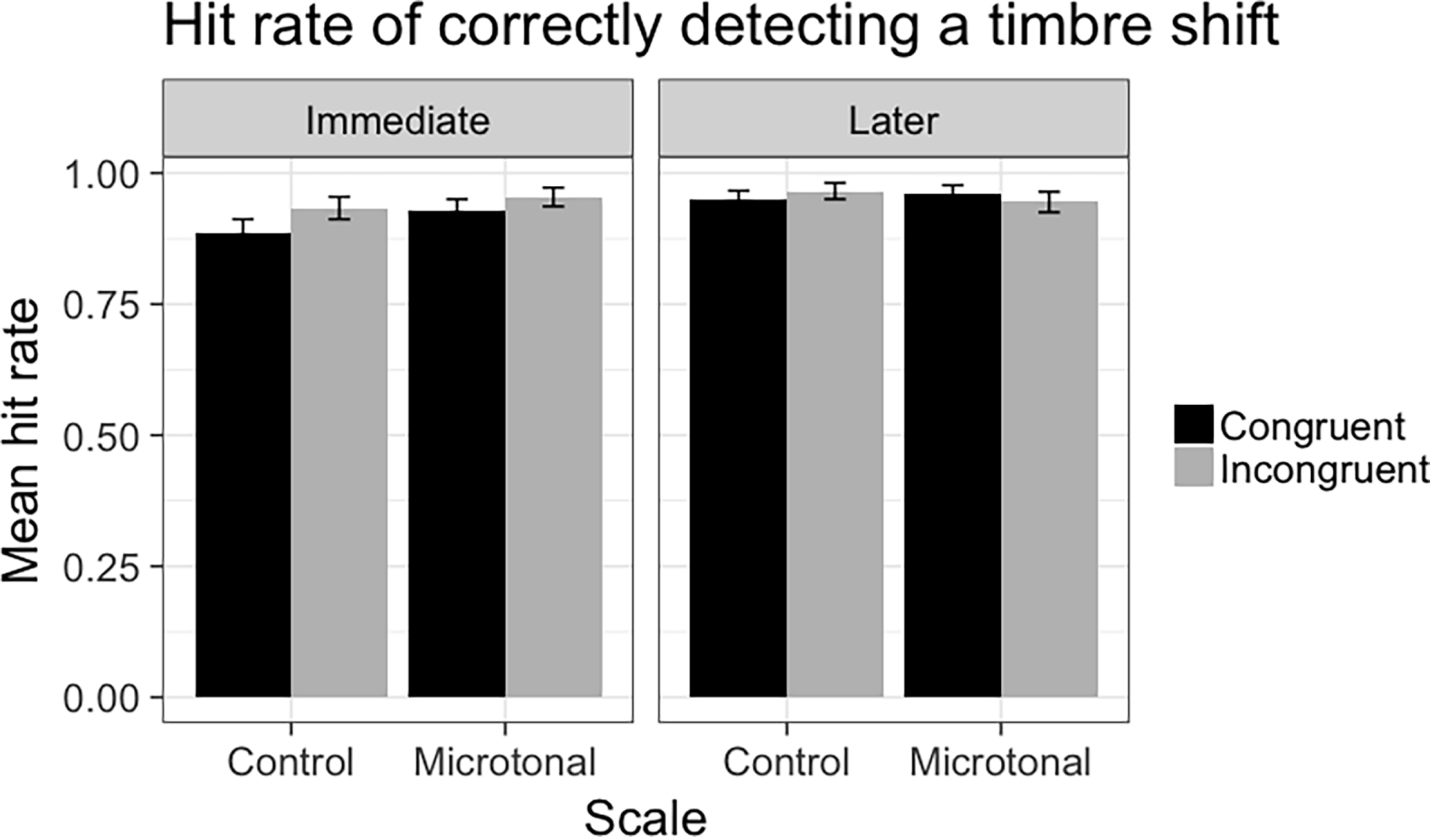
Mean hit rate in correctly detecting a timbre shift across scales and congruency conditions.

To assess participants’ accuracy in timbre shift detection and examine how it might be affected by the factors influencing RT, we analysed the Hit rate of correctly detecting the timbre shift across testing conditions, as displayed in Fig 8. As mentioned earlier, the overall hit rate is very high and it appears that the overall hit rate was higher in incongruent and later phase, which we will analyse it in the model below.

The model compared data from Congruent and Incongruent conditions, and Test Phases, to provide a complete analysis of the impact of Scale. Results showed that hit rate was significantly higher in the Incongruent condition and in the Later test. It was also significantly higher in the Microtonal condition. In addition, hit rate increased significantly over time (Trial) and when the timbre shift occurs later in the melody.

We then conducted a mixed effects model of the hit rate data from the Incongruent condition only. The purpose of this is to test besides the fixed effects above, whether Pitch Distance would be a significant factor. Results showed that Scale and Congruency did not influence the hit rate significantly. However, when the Pitch Distance increases, the hit rate decreases. In addition, hit rate did not vary extensively among participants across trials, based on the random effects analysis.

### False alarms in trials with a randomly located incongruent tone

**Fig 9:**
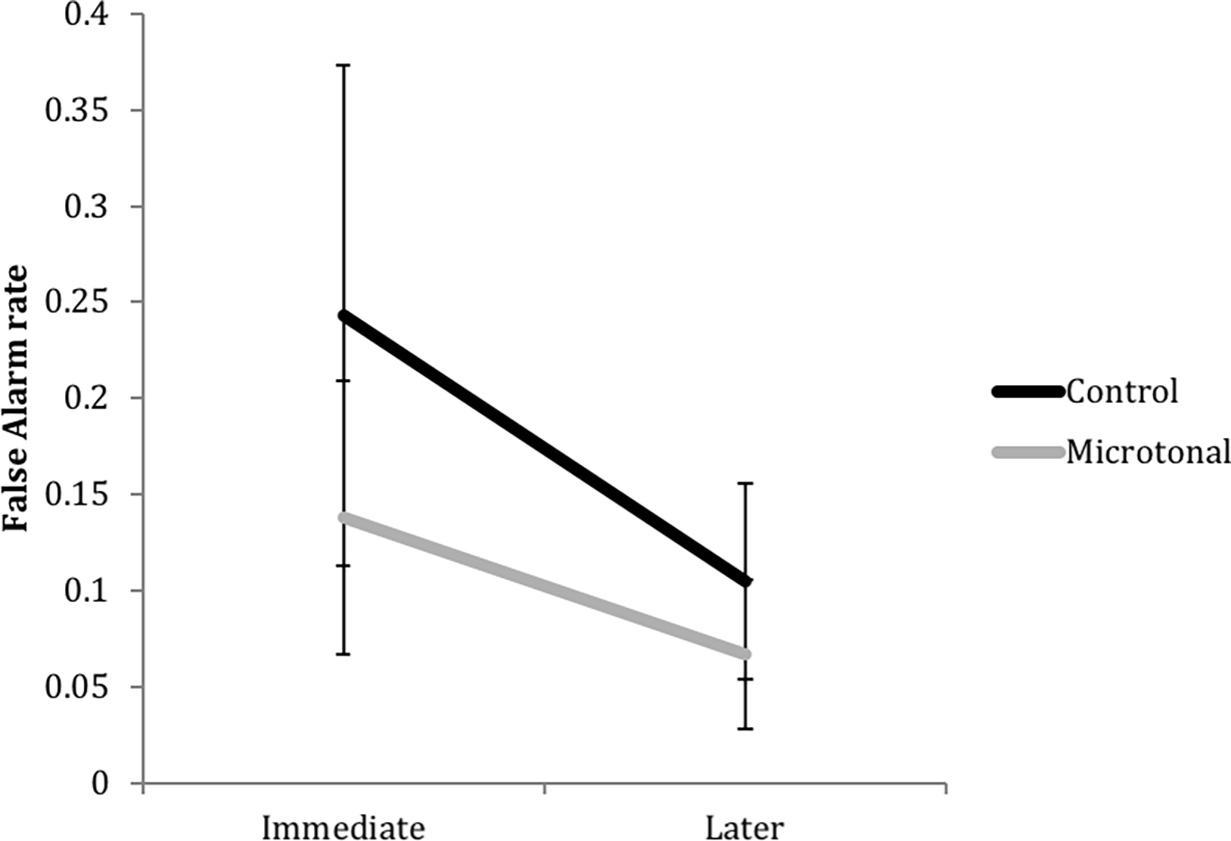
Mean false alarm rate in trials with no timbre shift but a randomly located incongruent tone, between scales and test phases.

We analysed the low false alarm rates in the trials where there was no timbre shift but participants responded as if there was, due to an randomly embedded incongruent tone. If participants perceive it as incongruent and hence as a strong predictor of the timbre shift, they might make a false alarm response after hearing the incongruent tone as if they were anticipating a timbre shift to follow. With Scale and Test Phase as the fixed effects, results showed that false alarm rate was lower in the Microtonal condition and in the Later Test, but there was no significant interaction between Scale and Test Phase (see Fig 9). A summary of results from the mixed effects models is presented in Table 2.

**Table 2:** Summary of results from the mixed effects models.

	Scale (vs Microtonal)	Congruency (vs Incongruent)	Scale*Congruency	Test Phase (vs Later)	Scale*Test Phase	Trial	Tone Position	Pitch Distance	RMSE
RT in all	-21.47 [-.32.80, -10.15]***	-48.97 [-60.86, -37.08]***	30.76 [14.05, 47.48]***	-42.13 [-50.46, -33.79]***	n.s.	n.s.	-17.39 [-20.41, -14.37]***	N/A	139.28
RT in Cong	-34.23 [-49.90, -18.57]***	N/A	N/A	-69.72 [-85/33, -54.11]***	23.67 [1.81, 45.52]*	21.07 [1.85, 40.29]*	-21.05 [-25.06, -17.03]***	N/A	133.85
RT in Incong	n.s.	N/A	N/A	-27.56 [-45.53, -9.59]**	n.s.	n.s.	-12.87 [-17.44, -8.31]***	n.s.	141.91
Hit rate in all	.02 [.002, .03]*	.01 [.001, .03]*	n.s.	.03 [.02, .04]***	n.s.	-.02 [-.03, -.004]*	.008 [.003, .01]**	N/A	.23
Hit rate in Incong	n.s.	N/A	N/A	n.s.	n.s.	n.s.	n.s.	-.08 [-.12, -.03]***	.21
False Alarms	-.07 [-.13, -.02]*	N/A	N/A	-.10 [-.16, -.05]***	n.s.	N/A	N/A	N/A	.12

Values are coefficient of the fixed effect [95% confidence interval]. P-value are represented by * (< .05), ** (< .01), *** (< .001). n.s. = not significant and N/A = Not applicable.

* between fixed effects is the interaction term of those effects. RMSE = Root-mean-square error (or square root of the variance of the residuals) of the model, which indicates the absolute fit of the model to the data (mean RT in correct timbre shift detection per participant can be found in S1 Data).

We had run separate ANOVAs on the same dataset and the results were consistent with those from the mixed-effects models, though weaker and less informative. The mixed effect models quantitated the impact of each fixed factor (not just based on the t value), and allowed us to examine factors that could not be accommodated in the ANOVA analysis, such as incongruent tone positions, trials, and random factors. Dealing also with the random effects, these models considerably strengthen the interpretations available from the ANOVAs.

## Discussion

This experiment examined the incidental learning of a novel, microtonal musical scale through a short period of exposure, with an experimental paradigm that we adapted from the concepts of masked priming [36] and statistical learning of event probabilities [18]. The learning was ‘incidental’ as participants were not informed of the real purpose of the task (since their sensitivity to the timbre shifts was in fact unrelated to our research question). Nonetheless, through engaging in the task, participants attended to each relative pitch of the microtonal scale repeatedly and became familiar with the pitch intervals of the scale. By applying the statistical manipulation of the tone before the timbre shift, we were able to measure listeners’ sensitivity to incongruent intervals within a musical context that they have just been exposed to. We found that after about ten minutes of listening to melodies from the microtonal scale, listeners without any musical training had become sensitive to the pitch intervals of the scale and were able to differentiate them from the pitch intervals of the diatonic scale, a musical scale to which they are enculturated. Learning was further validated by the performance in trials where a randomly located incongruent tone was embedded in melodies. When an incongruent tone was presented without being followed by a timbre shift, listeners sometimes make an immediate response after the tone (false alarms), as if they were anticipating a timbre shift to follow. These results of successful learning are consistent with prior research on artificial grammar learning of musical structure as well as statistical learning of musical sequences [16,18,34]. With a similar, short timeframe of learning as [46], a study that investigated the learning of distinctive modes in North Indian music among non-musicians, the current findings have contributed further with a microtonal scale that is not originated from any particular culture. Recently, with the same microtonal scale but a different group of non-musicians, we examined whether they could learn the tonal hierarchy of the scale. As expected, we found that they were able to learn different patterns of event frequency (frequency of appearance in the presented melodies) of members of the scale [47]. Learning was implied by the positive correlation observed between the event frequency of each member of the scale and their received goodness of fit ratings. Together with the current findings, both of our works suggest that learning the pitch structure and tonal hierarchy of a microtonal scale in less than 30min is possible among musically untrained individuals.

The expected learning in this study is considered challenging. Besides acquiring the information about the pitch intervals of the microtonal scale, participants were expected to learn the statistical association between tones and the timbre shifts. The association changes during the test phase to make the incongruent tone a better predictor of the timbre shift than the congruent tone, while p(TS|Cong) gradually declines. While participants were not instructed to attend to this relationship, their changing reaction time to the shift shows that they have learned this association, which is consistent with our hypothesis and prior findings on successful statistical learning of prime-target relationships [32,48]. When the incongruent tone has become a promising cue to the timbre shift, participants develop expectations of this association, reflected by their speeded reaction time. At the same time, RT became significantly longer in the Congruent condition overtime due to the decreasing p(TS|Cong). This observation is consistent with the semantic priming in lexical decision task and the cost and benefit analysis we discussed earlier [38,39]. And because of this, when the timbre shift did not occur after the incongruent tone in some of the trials, participants could make an accidental key press (false alarm) as this has become automatic. However, if participants had not perceived the incongruent tone as being a non-member of the scale, we would not have found this statistical association. This supports the view that our paradigm provides a robust measurement of learning.

Unlike past studies which often focused on testing the learning of stimuli from one tuning system (commonly in 12-tet) [41,49], the current experiment has made a comparison of such learning between diatonic and microtonal scales. Our findings reflect participants’ level of knowledge of our two different music systems, based on the automatic false alarms they made after hearing the incongruent tone. Although we find comparable reaction time data between the Control and the Microtonal conditions, more false alarms were made in the Control condition. As false alarms here imply one’s sensitivity to the incongruent tones, participants were better at detecting them in their familiar context than the newly exposed context. This finding is accordant with theories on musical schema, expectancy (e.g. schematic expectations by [31,50]), or processing fluency [51,52]). Such literature proposes that we are better at processing information in a musical context that we are familiar with, and we use our existing musical knowledge to process the incoming music, which can be of the same or a different music system. As participants’ level of diatonic or microtonal scale knowledge (such as musical expertise or other forms of prior exposure) might influence the learning of a microtonal scale, a follow-up study was conducted with musicians who are familiar with the 12-tet system and those with multiple microtonal systems [47]. We found that surprisingly, microtonal musicians did not show superior learning over 12-tet musicians. In fact, their overall RT was significantly slower than the 12-tet musicians, and they did not show the expected response pattern in the Incongruent condition. We suspected that due to their prior exposure of and practice with many microtonal systems, incongruent pitch intervals might not appear to be as deviant as we expected them to seem. Furthermore, those microtonal musicians we recruited are also computer music composers who have great interests in timbre. Therefore, they reported paying a lot of attention to the timbre shift itself, which might have slowed down their responses. Thus, the current paradigm might not be an appropriate approach to measure learning ability of musicians with a variety of musical experience such as multi-instrumentalists who play music in different tuning systems.

Although what makes a tone incongruent is based on the deviant pitch interval it has generated with the neighbouring tones, we did not find any statistically significant influence of pitch interval difference on reaction time. However, hit rate in the Incongruent condition decreased significantly when the pitch interval difference is bigger between the original and the incongruent intervals. In other words, when the incongruence becomes more obvious to the listeners, they are more likely to miss the timbre shift. However, if they manage to catch it and respond, their reaction time is faster than that in the congruent condition. As yet, we have no strong explanation for this, though it is somewhat akin to the phenomenon of categorical perception.

## Conclusion

To conclude, the current paper found that musically untrained listeners could learn a novel, microtonal musical scale rapidly through a new paradigm we designed. This paradigm is validated to be robust in measuring the incidental learning of musical pitch structure, and we believe it can be used in statistical learning experiments in other modalities. Listeners develop preferences for music that they are familiar with, and musical preference relates to music-evoked emotional responses. If learning a new music system does not take very long, listeners might form a positive musical preference for such music over a short period too. We believe this finding has implications not only for experimental psychology in music but also for the creative music industry.

## Supporting information

S1 Table Gold-MSI score of each participant. (DOCX)

S1 Text Explanation of mixed-effects models. (DOCX)

S1 Data Mean reaction time in correct timbre shift detection per participant. (XLSX)
