# Supplementary material for: Learning unfamiliar pitch intervals: A novel paradigm for demonstrating the learning of statistical associations between musical pitches
Source: PLoS One. 2018 Aug 30;13(8):e0203026. doi: 10.1371/journal.pone.0203026 (PMC6117015; doi:10.1371/journal.pone.0203026)
Supplement: S1 Table — (DOCX) [file pone.0203026.s001.docx]

S1 Table

Table. Gold-MSI score of each participant

| ID | Active Engagement | Perceptual Abilities | Musical Training | Emotions | Singing Abilities | General Sophistication |
| --- | --- | --- | --- | --- | --- | --- |
| 1 | 41 | 51 | 13.5 | 35 | 35 | 82.5 |
| 2 | 36 | 44 | 18 | 34 | 31 | 77 |
| 3 | 15 | 32 | 2 | 22 | 22 | 37 |
| 4 | 22 | 54 | 3 | 27 | 24 | 52 |
| 5 | 21 | 38 | 7 | 31 | 24 | 53 |
| 6 | 21 | 38 | 8 | 23 | 21 | 50 |
| 7 | 29 | 36 | 3 | 28 | 16 | 44 |
| 8 | 30 | 45 | 4 | 33 | 33 | 60 |
| 9 | 24 | 25 | 6 | 33 | 26 | 48 |
| 10 | 19 | 45 | 5 | 32 | 20 | 43 |
| 11 | 26 | 43 | 11 | 32 | 25 | 56 |
| 12 | 33 | 49 | 13 | 28 | 23 | 66 |
| 13 | 27 | 46 | 13 | 32 | 35 | 71 |
| 14 | 20 | 45 | 2 | 32 | 8 | 34 |
| 15 | 28 | 42 | 8 | 33 | 29 | 58 |
| 16 | 21 | 33 | 8.5 | 22 | 15 | 40.5 |
| 17 | 26 | 45 | 4 | 25 | 19 | 49 |
| 18 | 26 | 45 | 6 | 36 | 27 | 56 |
| 19 | 25 | 41 | 5 | 38 | 21 | 48 |
| 20 | 25 | 36 | 2 | 29 | 25 | 45 |
| 21 | 13 | 36 | 5 | 18 | 9 | 28 |
