# Supplementary material for: Learning unfamiliar pitch intervals: A novel paradigm for demonstrating the learning of statistical associations between musical pitches
Source: PLoS One. 2018 Aug 30;13(8):e0203026. doi: 10.1371/journal.pone.0203026 (PMC6117015; doi:10.1371/journal.pone.0203026)
Supplement: S1 Text — (DOCX) [file pone.0203026.s002.docx]

# S2 Text

## Explanation of mixed-effects models

Mixed-effects modelling is a regression analysis of two types of effects: fixed and random effects. Predictions based on the intercept and coefficients of the fixed effects are expected to be generalizable to the population, such as gender or age group. Fixed effects can represent the impact of the independent variables or the experimental task experience that we manipulate, such as control versus experimental group and types of stimuli, the number of trials, etc. Intercepts and slopes of the random effects, however, can vary across sub-groups or individuals in a set of participants, such as differences in intercepts for task performance between participants. A mixed-effects model tests the significance of fixed effects as predictors and whether the standard deviation of the predicting value contributed by the random effects is significant. If it is, this means the predicted value (e.g. reaction time) is influenced by both the fixed and random effects and it could vary systematically across individuals or divisions of the sample. A likelihood ratio test can inform us about whether the model with the random-intercepts offers a significantly better prediction (e.g. a lower percentage of unexplained residual) than a linear regression model with fixed effects only.

A linear fixed-effects only regression model with two fixed effects:

y_i_ = β_0_ + β_1_x_1i_ + β_2_x_2i_ + ε_i_

where y is the predicted score of participant i, β represents the fixed effect. β_0_  is the intercept term, while β_1_ and β_2_ refers to the coefficients of the first (x_1_) and second fixed effects (x_2_) that are predictors of y. ε is a residual error term.

The formula of the mixed-effects model is in a similar form, but with the additional random intercept u_0_. j represents the variability between the sub-groups or individual participants defined in the random effects:

y_ij_ = β_0_ + β_1_x_1ij_ + β_2_x_2ij_ + u_0j_ + ε_ij_

Sometimes the slope of the random effects is interacted or correlated with one of the fixed effects. For instance, performance across trials (fixed effects) could vary systematically between participants, where participants who performed worse in the earlier trials tend to have a higher improvement rate than those who performed well when they first started doing the task. In this case, a mixed model would also include random slopes of ‘inter-subject variability’ (u_1j_) on predictor x_1_ (trial number in this case):

y_ij_ = β_0_ + β_1_x_1ij_ + β_2_x_2ij_ + u_0j_ + u_1j_x_1ij_ + ε_ij_

Our aim is to derive models driven by the design of the experiment with fixed and random effects as candidate predictors and to select those that fit our data best (as judged by BIC and RMSE, and finally likelihood ratio tests). Random effects analysis also allows the exploration of individual differences between participants, which cannot be explained by ANOVA: ANOVA assumes the variance between participants within each experimental group is homogeneous. Past papers have argued that linear mixed-effects models offer a better approach than univariate ANOVA or ordinary least squares regression and they have been widely used in longitudinal and linguistics studies (e.g. ,1–3).

1. Baayen RH, Davidson DJ, Bates DM. Mixed-effects modeling with crossed random effects for subjects and items. J Mem Lang. 2008;59(4):390–412.

2. Cunnings I. An overview of mixed-effects statistical models for second language researchers. Second Lang Res [Internet]. 2012;28(3):369–82. Available from: http://slr.sagepub.com/content/28/3/369.full.pdf

3. Wainwright PE, Leatherdale ST, Dubin JA. Advantages of mixed effects models over traditional ANOVA models in developmental studies: A worked example in a mouse model of fetal alcohol syndrome. Dev Psychobiol [Internet]. 2007 Nov [cited 2016 Nov 4];49(7):664–74. Available from: http://doi.wiley.com/10.1002/dev.20245
